# Design and characterization of dual drug delivery based on *in-situ* assembled PVA/PAN core-shell nanofibers for wound dressing application

**DOI:** 10.1038/s41598-019-49132-x

**Published:** 2019-09-02

**Authors:** Davood Kharaghani, Parastoo Gitigard, Hijiri Ohtani, Kyu Oh Kim, Sana Ullah, Yusuke Saito, Muhammad Qamar Khan, Ick Soo Kim

**Affiliations:** 1 Nano Fusion Technology Research Group, Division of Frontier Fibers, Institute for Fiber Engineering (IFES-, Interdisciplinary Cluster for Cutting Edge Research) ICCER, Shinshu University, Tokida 3-15-1, Ueda, Nagano 386-8567 Japan; 2 Department of Fiber System Engineering, Dankook University, Gyeonggi-do, Republic of Korea

**Keywords:** Nanostructures, Drug delivery

## Abstract

Core-shell nanofibers with the ability to carry multiple drugs are attracting the attention to develop appropriate drug delivery systems for wounds dressing applications. In this study, biocompatible core-shell nanofibers have been designed as a promising dual-drug carrier with the capability of delivering both water-soluble and organic solvent-soluble drugs simultaneously. With the aim of fabricating the core-shell nanofibers, the dipping method has been employed. For this propose, core nanofibers made from polyvinyl alcohol (PVA) were immersed in various concentrations of polyacrylonitrile (PAN) and cross-linked by dipping into ethanol. Diclofenac sodium salt (DSs) and gentamicin sulfate (GENs) have been loaded into the core and shell nanofibers as models of the drug, respectively. The morphology study of core-shell nanofibers showed that the concentrations between 1% w/w up to 2% w/w PAN/GENs, with deep penetration into the internal layers of PAV/DSs nanofibers could lead to the core-shell structure. The cytotoxicity results showed the competency of designed core-shell nanofibers for wound dressing application. Also, the release profile exhibits the controllable behavior of drug release.

## Introduction

Nanofibers with simulating the extracellular matrix (ECM)^1,2^, are showing the appropriate potential for design the wound dressing and drug delivery systems^3^. Different kind of nanofibers, such as core-shell nanofibers for drug delivery applications, have been employed as an effective and innovative architecture^4–6^. In the last years, dual drug delivery systems based on water-soluble and organic solvent-soluble drugs remaining the challenges when specific target required multiple medications^7–9^. Among the advanced methods, appropriate drug delivery systems have been designed based on co-axial or core-shell electrospun nanofibers^10,11^. However, the envisaged issues to prepare core-shell nanofibers are adjusting the supplied electrical voltage and polymers viscosity to obtain the uniformed nanofibers^12,13^. Therefore, designing the facile methods as well as dipping methods could help to fabricate the core-shell nanofibers for dual drug delivery system^14–17^.

On the other hand, for designing the dual drug delivery systems based on water-soluble and organic solvent-soluble drugs, the polymers should meet the requirements such as biocompatibility and solubility in water and organic solvent. PVA is a biocompatible and hydrophilic polymer, which recently has been used as an undertaking material for scaffold nanofibers preparations regarding tissue engineering and drug delivery applications after cross-linking via glutaraldehyde (GA)^18–21^. However, polyacrylonitrile (PAN) is soluble in the organic solvents, and nanofibers from PAN in addition to carrying the organic solvent soluble drugs are showingthe biocompatibility against the fibroblast cells^14,22^.

**Figure 1:**
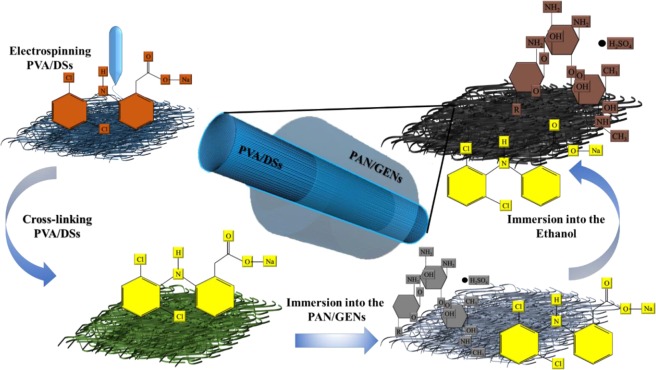
Illustration skims of core-shell nanofiber for dual simultaneous drug delivery.

Therefore, we developed a dual drug delivery system for wound dressing application based on PVA-PAN core-shell nanofibers with the ability to caring DSs and GENs as anti-inflammation and antibiotic drugs. The DSs and GENs are well-known drugs as anti-inflammatory and antibiotic to reducing inflammatory for the regeneration of wound. Figure 1 shows the method in brief.

## Results and Discussion

### The morphology study

**Figure 2:**
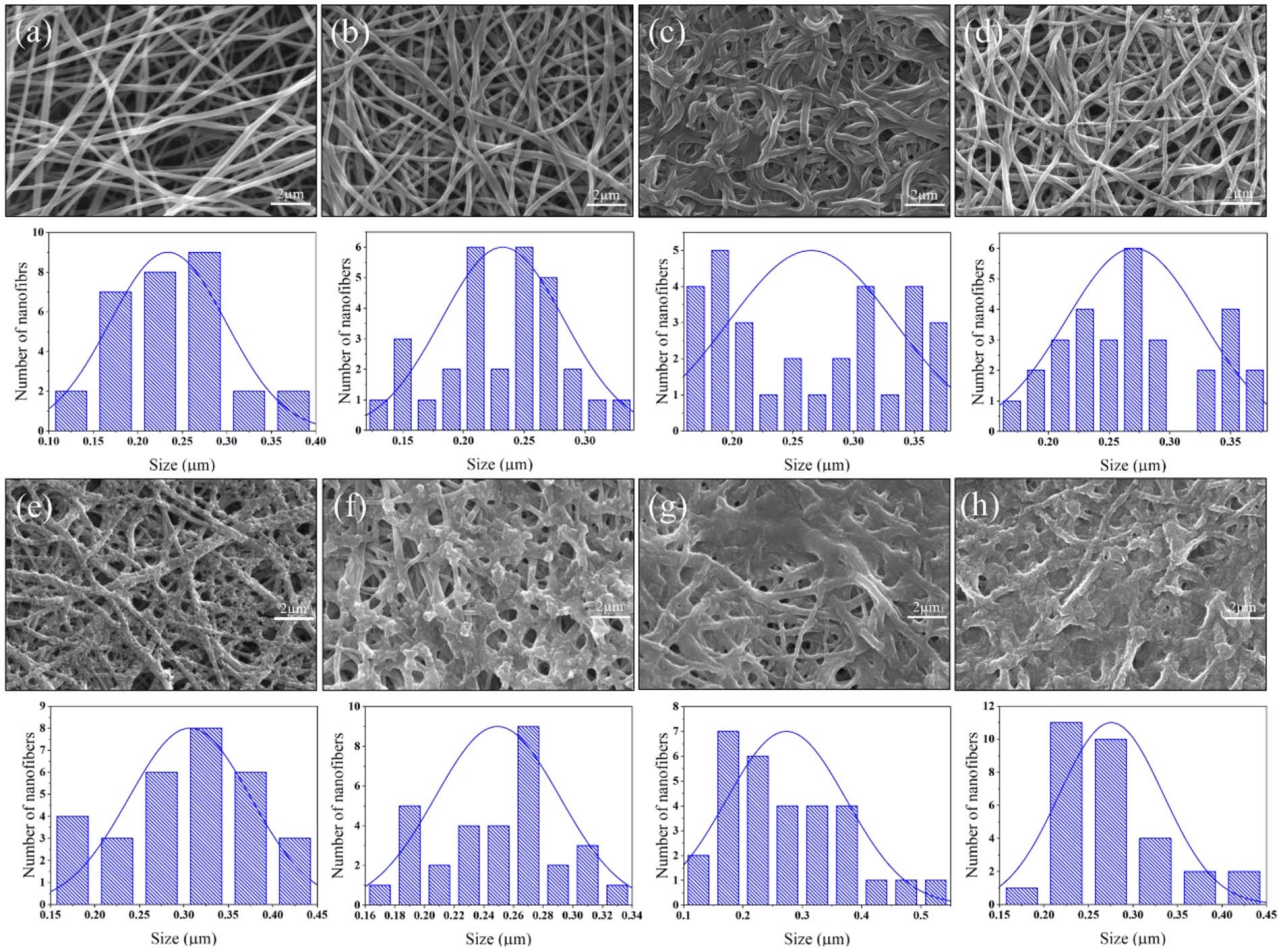
SEM of PVA/DSs nanofibers before crosslinking: (**a**) PVA/DSs nanofibers after crosslinking: (**b**) and cross-linked PVA/DSs after immersing in 0.5% w/w PAN/GENs: (**c**) 1% w/w PAN/GENs: (**d**) 2% w/w PAN/GENs: (**e**) 3% w/w PAN/GENs: (**f**) 4% w/w PAN/GENs: (**g**) and 5% w/w PAN/GENs: (**h**).

The scanning electron microscopy (SEM) was used for the morphology study of the prepared nanofibers in all steps, as shown in Fig. 2. It was observed that PVA/DSs with a concentration of 5 mg/mL qualified without any bead and compared with similar structures where bead formation has been explained^23^. From the morphology study of nanofibers, it was obtained that GA did not have a significant effect on the morphology of nanofibers after cross-linking as presented in Fig. 2(b) and described in the previous literature^24^.

To prepare the final core-shell nanofibers, five different concentrations of PAN solution containing GENs were applied. The SEM images showed that the morphology of PVA/DSs nanofibers affected by 0.5% w/w of PAN/GENs solution due to the high concentration of dimethylformamide (DMF). On the other hand, increasing the concentration of PAN/GENs to more than 2% w/w showed that the solutions with blocking the pores on the surface caused to prevent the PAN/GENs solution penetration into the PVA/DSs nanofibers mat.

The mean diameter for the final nanofibers has been calculated as below in Table 1.

**Table 1:** The mean diameter for the final nanofibers. In all cases SD < 0.005 μm.

samples	Mean diameter (μm)
Uncross-linked PVA/DSs	0.233
Cross-linked PVA/DSs	0.232
PVA/DSs-PAN/GENs 0.5%	0.265
PVA/DSs-PAN/GENs 1%	0.270
PVA/DSs-PAN/GENs 2%	0.306
PVA/DSs-PAN/GENs 3%	0.249
PVA/DSs-PAN/GENs 4%	0.273
PVA/DSs-PAN/GENs 5%	0.275

However, the results showed that submersing the nanofibers into the PAN/GENs solution and coating the surface of PVA/DSs electrospun nanofibers, has changed the mean diameter of nanofibers as presented in Table 1 and Fig. 2.

**Figure 3:**
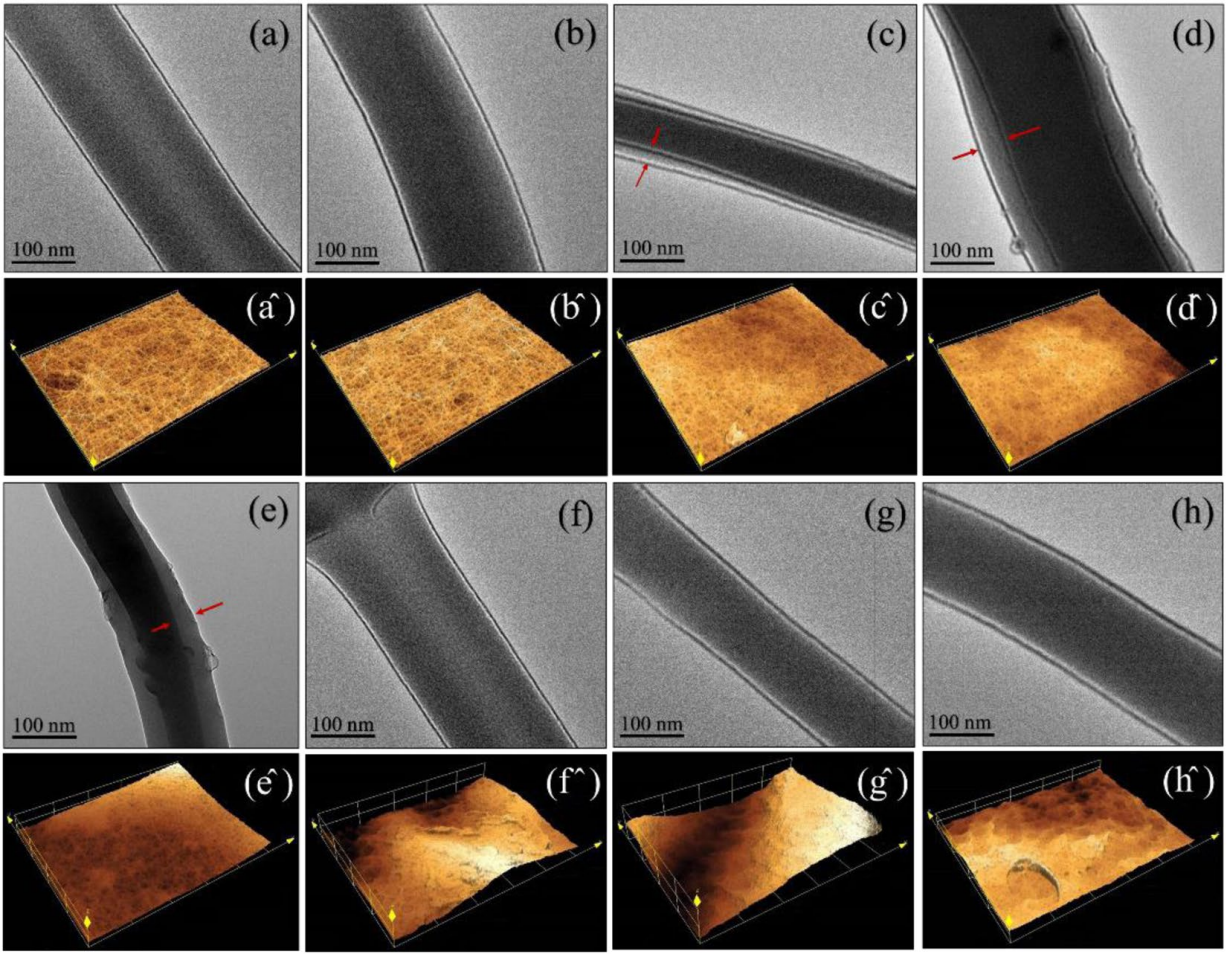
The TEM images and laser scanning confocal microscopy of uncross-linked PVA-DSs: (**a**) and (a^^^), cross-linked PVA-DSs: (**b**) and (b^^^), PVA-DSs/PAN-GENs was coated with 0.5% w/w PAN-GENs (**c**) and (c^^^), PVA-DSs/PAN-GENs was coated with 1% w/w PAN-GENs (**d**) and (d^^^), PVA-DSs/PAN-GENs was coated with 2% w/w PAN-GENs (**e**) and (e^^^), PVA-DSs/PAN-GENs was coated with 3% w/w PAN-GENs (**f**) and (f^^^), PVA-DSs/PAN-GENs was coated with the 4% w/w PAN-GENs (**g**) and (g^^^), PVA-DSs/PAN-GENs was coated with 5% w/w PAN-GENs (**h**) and (h^^^).

The transmission electron microscopy (TEM) was used for observing the inner structural nanofibers (Fig. 3). The TEM results are shown the interface between the core and shell polymers in Fig. 3(c–e) related to PAN/GENs solutions with concentrations of 0.5%, 1%, and 2% w/w. However, with increasing the concentration of PAN/GENs solution to more than 2% w/w, core-shell structures did not form due to blocking the porosity with high concentrations of PAN/GENs solutions. Furthermore, core-shell nanofibers prepared from PAN/GENs with concentrations from 1% to 2% w/w were showed relevant results among the other concentrations. Also, from the laser scanning confocal microscopy (LSCM) was confirmed that the morphology of surface changed when the concentration of PAN/GENs has increased to more than 2% w/w. These results demonstrated that the dipping method was suitable to prepare the core-shell nanofibers structure.

Preserving the structure of nanofibers are essential due to simulating the extracellular matrix (ECM), especially for biomedical applications. Therefore, in the current research emphasis was on preserving the original structures of nanofibers after achieving the final core-shell structure. The SEM and LSCM images results showed that the structure of nanofibers and porosity was preserved after coating the nanofibers by PAN/GENs solution with concentrations of 1% and 2% w/w and followed by TEM monitoring. Also, the TEM images showed that the best core-shell nanofibers were formed by using 1% and 2% w/w of PAN/GENs solution successfully.

**Figure 4:**
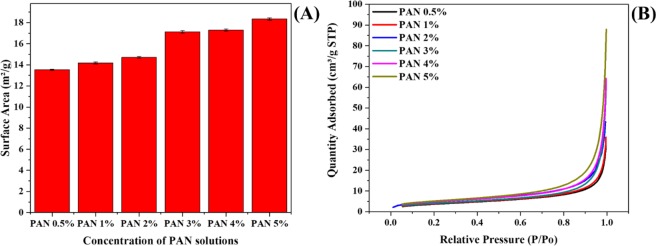
Surface to area plot of core-shell nanofibers prepared by PAN/GENs solutions with the range of 0.5% to 5% w/w: (**A**) and amount of nitrogen adsorption by with the range of 0.5% to 5% w/w PAN/GENs solutions in varying relative pressure: (**B**).

The Bunner-Emmett-Teller (BET) was used to evaluate the PAN/GENs penetration into the PVA/DSs nanofibers. As shown in Fig. 4(A), from the results, obtained that by increasing the concentration of PAN/GENs, the ratio of surface to the area was gradually increased. As shown in Figs 2 and 3, It seems that when 0.5% to 2% w/w of PAN/GENs solutions have been used to form the core-shell structures the size distribution of nanofibers was increased due to PAN/GENs penetration into the PVA/DSs. Therefore, increasing the size distribution lead to decreasing the surface to the area and pore size. However, with the increasing PAN/GENs concentration, polymerization of PAN occurred on the surface of PVA/DSs and caused to block the surface pores for PAN/GENs penetration. Therefore, the internal nanofibers preserved from coating occurrences and caused to increase the surface area. Decreasing the surface area was a clear indication towards higher penetration of PAN/GENs into the PVA/DSs nanofibers when PAN/GEN with the concentrations of less than 2% w/w have applied.

Also, Fig. 4(B) is shown a plot between relative pressure and the amount of nitrogen adsorption on the surface of core-shell nanofibers. From the results, it was assumed that with increasing relative pressure, the amount of liquid nitrogen also increased. Also, results showed that increasing trend in adsorption could translate to a higher surface area viability for nitrogen absorption by core-shell nanofibers prepared by PAN/GENs with the concentration of more than 2% w/w. From the results confirmed that the PAN/GENs solutions with the concentrations of less than 2% could penetrate homogeneously and preserve the original structure of nanofibers.

### FT-IR-ATR and XPS

**Figure 5:**
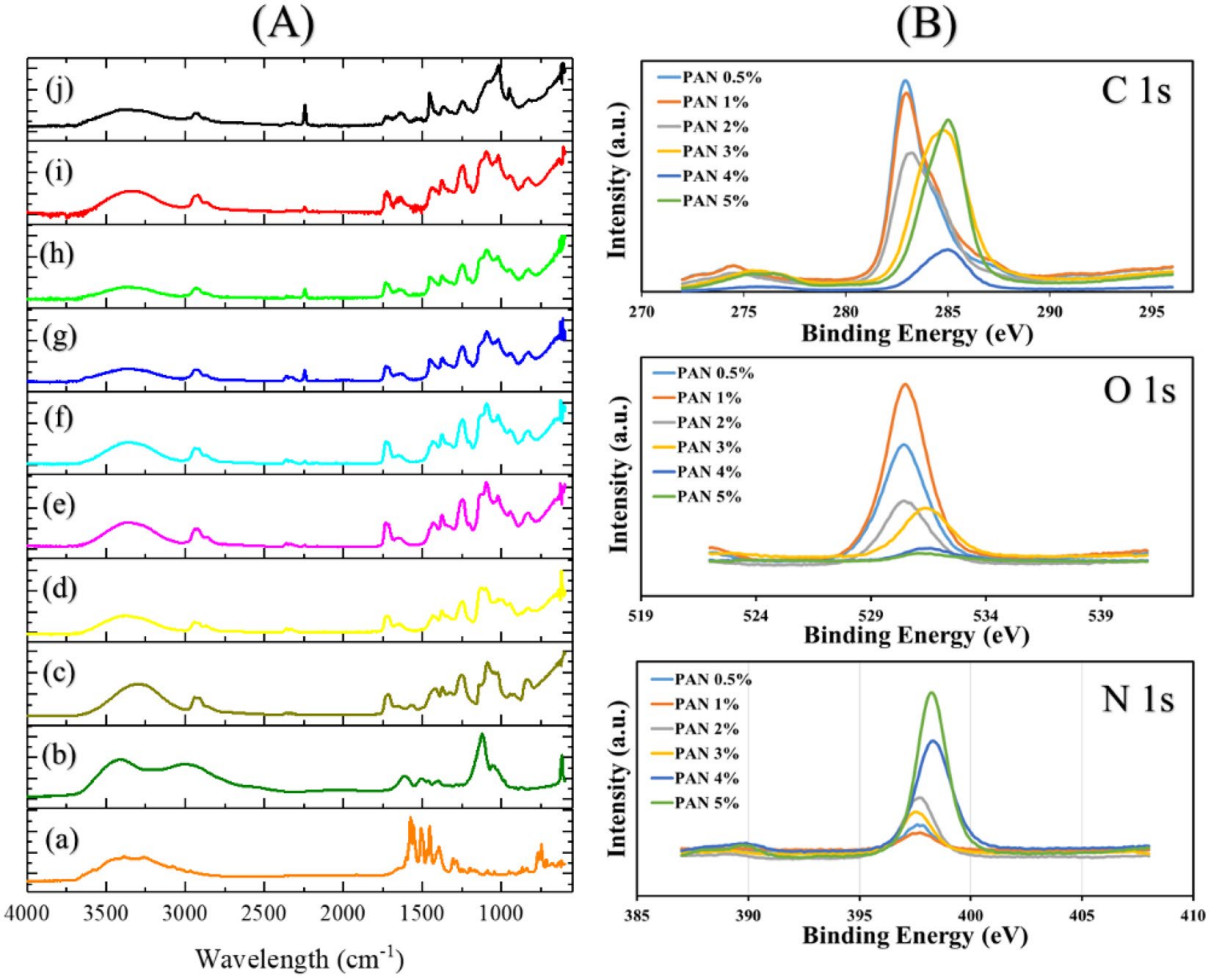
The FT-IR-ATR (**A**) from diclofenac sodium salt: (a) Gentamicin sulfate: (b) uncross-linked PVA/DSs: (c) cross-linked PVA-DSs: (d) PVA-DSs/PAN-GENs was coated with 0.5% PAN-GENs (e), PVA-DSs/PAN-GENs was coated with 1% PAN-GENs (f), PVA-DSs/PAN-GENs was coated with 2% PAN-GENs (g), PVA-DSs/PAN-GENs was coated with 3% PAN-GENs (h), PVA-DSs/PAN-GENs was coated with the 4% PAN-GENs (i), PVA-DSs/PAN-GENs was coated with 5% PAN-GENs (j). The XPS is presented in part (**B**) for carbon, oxygen and nitrogen species after developing the core-shell nanofibers with various concentrations of PAN/GENs solution from 0.5% w/w to 5% w/w.

Fourier-transform infrared spectroscopy (FT-IR) was carried out to investigate the chemical functional groups, as shown in Fig. 5. The spectrum of DSs has a band at 3391 cm^−1^ due to the presence of N-H. The band related to phenyl group appeared at 1580 cm^−1^ and substituted phenyl group stretch at 750 cm^−1^ in Fig. 5(a)^25^. The spectrum of pure GENs showed the band at 1615 cm^−1^, and 1512 cm^−1^ corresponding to an amide group. However, the peak at 1125 cm^−1^ represents the HSO_4_ group, 1068 cm^−1^ related to C-O-C and sharp band at 621 cm^−1^ appeared due to SO_2_ in the structure of GENs^26^. The PVA spectrum in Fig. 5(C), showed a wild band at 3298 cm^−1^ response to the presence of OH functional group that the intensity of the same peak was decreased and shifted to higher wavelength due to a chemical reaction between PVA and GA after crosslinking^24^. Therefore, from the results, as summarized in Table 2, it was concluded that the method for crosslinking the PVA nanofibers did not have a significant effect on the structure of loaded DSs due to the presence of phenyl group at 841 and 1650 cm^−1^. The functional groups related to PAN layer appeared at 2241 cm^−1^ due to C≡N group^13^.

**Table 2:** Summarized data from FT-IR.

Functional Group	Absorption(s) (cm^−1^)	Notes
N-H	3391	DSs
phenyl group	1580	DSs
phenyl group	750	Stretching DSs
Amide group	1615	GENs
Amide group	1512	Stretching GENs
HSO_4_	1125	GENs
C-O-C	1068	GENs
SO_2_	621	GENs
C≡N	2241	Stretching PAN
NH	3380	PAN
OH	3311	PVA
CH_2_	2922	CH stretching in CH and CH2 groups
C=O	1728	GA

In addition to FT-IR, the X-ray photoelectron spectroscopy (XPS) has utilized to characterize the chemical composition of the core-shell nanofibers prepared by using the various concentration of PAN/GENs from 0.5% w/w to 5% w/w. As shown in Fig. 4(B), by analyzing the C 1s, O 1s, and N 1s spectra, the core-shell nanofibers studied in terms of chemical characterization. The frequencies from C 1s shown two groups of the peak located at 283 eV and 285 eV were related to PAN/GENs with the concentrations of 0.5% w/w to 2% w/w and the concentrations more than 2% w/w respectively. However, the normal state of C 1s has been reported at 285 eV for PAN nanofibers^27^. On the other hand, the spectra from O 1s shown the two group of the peak at 530 eV and 531.5 eV as well as C 1s attribute to different spices of oxygen and carbon bonding^28^. From the N 1s spectra result, it was confirmed that increasing the concentration of PAN/GENs may lead to decreasing the intensity due to the aggregation of PAN/GENs polymer. While the distribution and penetration of PAN/GENs polymer related to the concentrations less than 2% w/w into the PVA/DSs nanofibers caused to increase the intensity in comparison to the high concentrations of PAN/GENs. The results showed that core-shell nanofibers formed with the maximum yield by using the concentrations from 0.5% w/w to 2% w/w.

**Figure 6:**
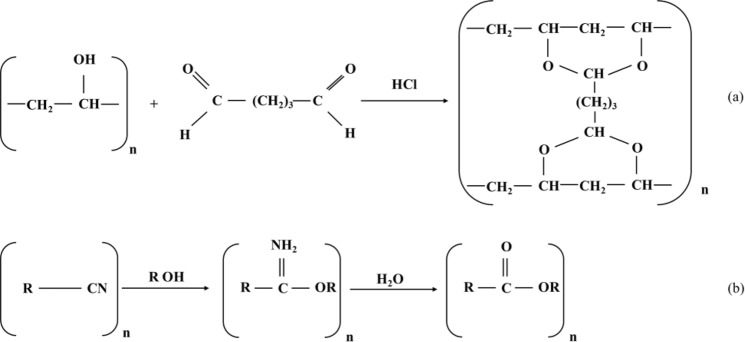
The crosslinking reaction between PVA and GA: (**a**) and the cross-linking reaction between PAN and ethanol: (**b**).

The crosslinking reactions for PVA and PAN have been shown in Fig. 6 as below:

As shown in the polymerization reaction in Fig. 6, the OH functional groups are playing a critical role as a cross-linking function. Also, the FT-IR results emphasized the hydroxyl groups from polymers, has participated in the nanofibers cross-linking reactions. However, the hydroxyl functional group may cause to adsorb and start the polymerization of PAN solution on the surface of PVA/DSs, and the polymerization was completed by immersion in the ethanol. Therefore, it seems that the current method may suitable for the nanofibers contain the OH functional groups such as cellulose and polyurethane.

### Thermal stability and *in-vitro* degradation

**Figure 7:**
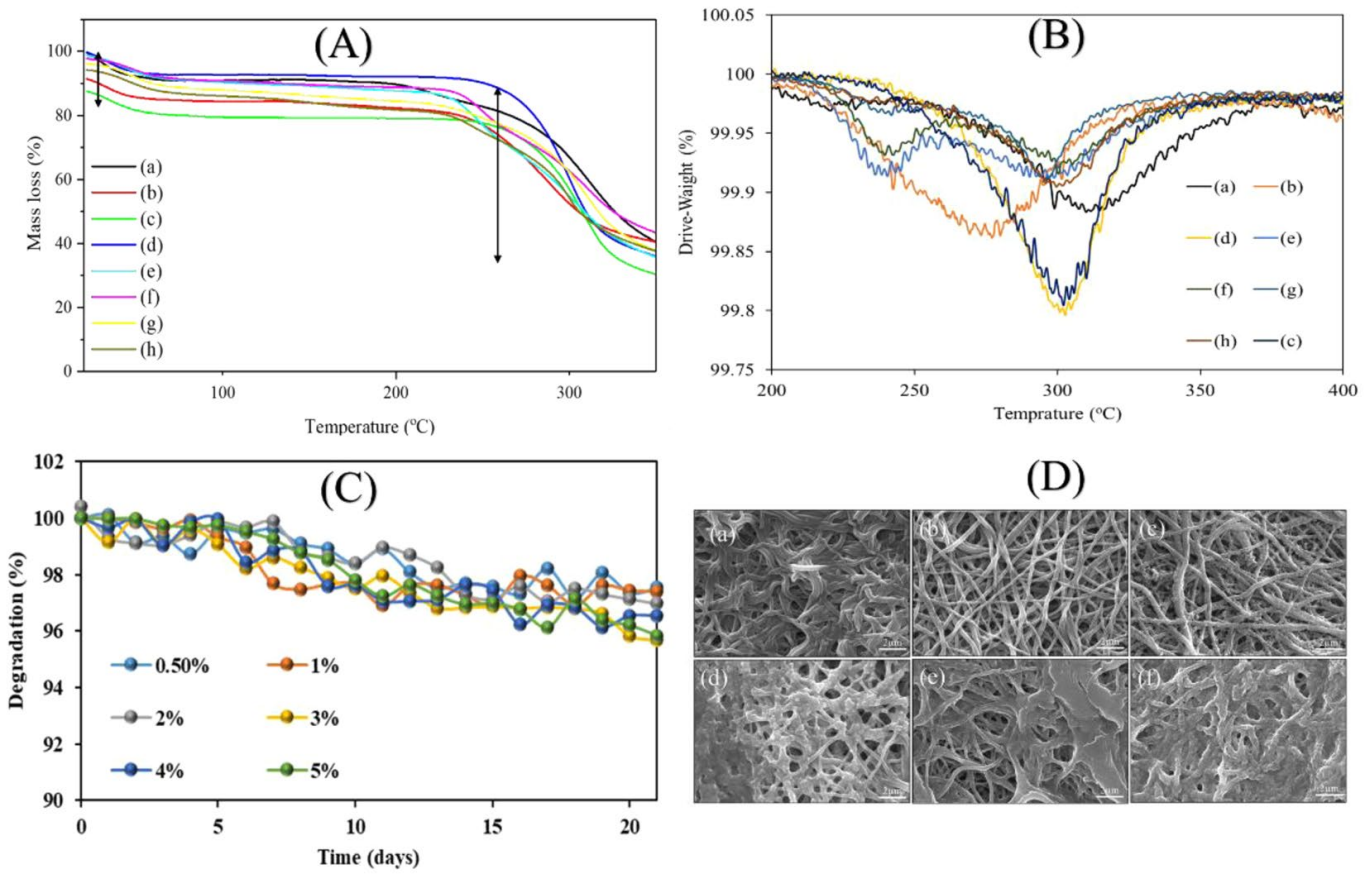
TGA thermos-gram: (**A**) DTGA thermos-gram: (**B**) of uncross-linked PVA/DSs: (a) cross-linked PVA-DSs: (b) PVA-DSs/PAN-GENs was coated with 0.5% PAN-GENs: (c) PVA-DSs/PAN-GENs was coated with 1% PAN-GENs (d), PVA-DSs/PAN-GENs was coated with 2% PAN-GENs (e), PVA-DSs/PAN-GENs was coated with 3% PAN-GENs (f), PVA-DSs/PAN-GENs was coated with the 4% PAN-GENs (g), PVA-DSs/PAN-GENs was coated with 5% PAN-GENs (h), degredation (**C**) and SEM images after 3 weeks of degradation (**D**) of PVA-DSs/PAN-GENs was coated with 0.5% PAN-GENs: (c) PVA-DSs/PAN-GENs was coated with 1% PAN-GENs (d), PVA-DSs/PAN-GENs was coated with 2% PAN-GENs (e), PVA-DSs/PAN-GENs was coated with 3% PAN-GENs (f), PVA-DSs/PAN-GENs was coated with the 4% PAN-GENs (g), PVA-DSs/PAN-GENs was coated with 5% PAN-GENs (h).

Thermogravimetric analyses (TGA and DTGA) spectra were studied to investigate the thermal stability of core-shell nanofibers. TGA and DTGA thermos-gram for different core nanofibers are shown in Fig. 7. The results from TGA thermos-gram (Fig. 7(A)) showed that all samples were containing the two-stages of decomposition. The first stage was related to the presence of humidity absorption, where it appeared at 90 °C. However, the main stage of decomposition related to PVA/DSs was started around 245 °C and completed around 341 °C^18^ and decomposition of nanofibers continued up to 350 °C. The final result has summarised in Table 3. From Table 3 it was concluded that cross-linking the PVA/DSs nanofiber could affect the thermal stability^29^. Decreasing the thermal stability could be related to morphology change of core-shell nanofiber with 0.5% w/w PAN/GENs concentration as showed in SEM results (Fig. 2(c)). To observe the trend and the difference between the samples, the DTGA was employed, as indicated in Fig. 6(B). From the results, it was confirmed that the concentrations more than 1% w/w PAN/GENs had different thermal behavior due to increasing the amount of bulk PAN on the surface of PVA/DSs nanofibers.

**Table 3:** Summarized data from TGA.

samples	The residue at 350 °C (%)
Uncross-linked PVA/DSs	40.53
Cross-linked PVA/DSs	40.53
PVA/DSs-PAN/GENs 0.5%	29.78
PVA/DSs-PAN/GENs 1%	36.26
PVA/DSs-PAN/GENs 2%	36.27
PVA/DSs-PAN/GENs 3%	38.35
PVA/DSs-PAN/GENs 4%	37.83
PVA/DSs-PAN/GENs 5%	37.5

For further investigation of *in-vitro* degradation, core-shell nanofibers were examined for 21 days in water at 37 °C. As reported in previous degradation studies related to PAN/GENs nanofibers, prepared core-shell nanofibers did not indicate significant degradation during the initial seven days^30^. From the results, as presented in Fig. 7(C), obtained that losing weight was started after seven days that it could be the appropriate degradation stage for the wound dressing applications.

### *In-vitro* cytotoxicity and cell adhesion

**Figure 8:**
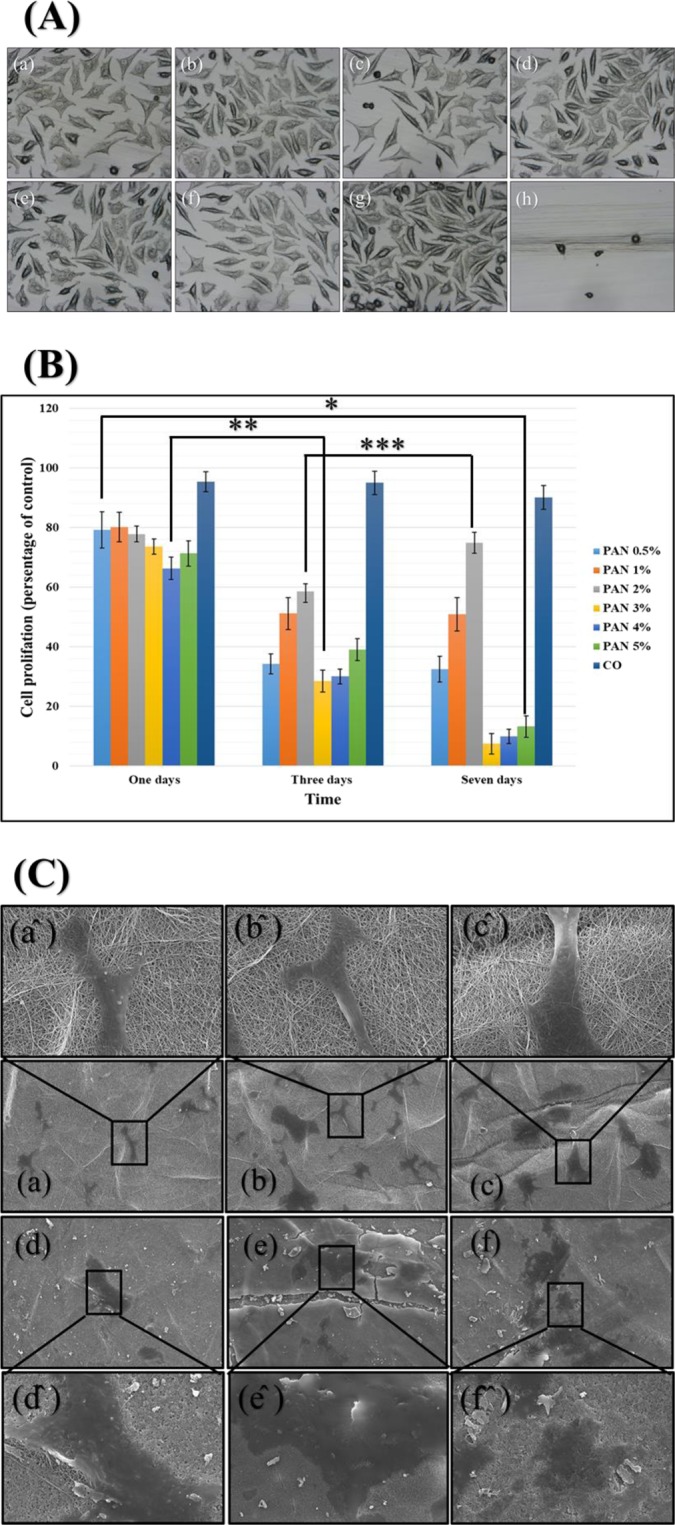
The cell toxicity (**A**): PVA-DSs/PAN-GENs 0.5% w/w PAN-GENs: (a) PVA-DSs/PAN-GENs 1% w/w PAN-GENs: (b) PVA-DSs/PAN-GENs 2% w/w PAN-GENs: (c) PVA-DSs/PAN-GENs 3% w/w PAN-GENs: (d) PVA-DSs/PAN-GENs 4% w/w PAN-GENs: (e) PVA-DSs/PAN-GENs 5% w/w PAN-GENs: (f) negative control: (g) and positive control: (h). The cell proliferation of samples after one, Three and seven days (**B**), (P-value * = 0.34, ** = 8.27E-06, *** = 0.36). The cell adhesion and cell migration (**C**) for PVA-DSs/PAN-GENs 0.5% w/w PAN-GENs: (a) PVA-DSs/PAN-GENs 1% w/w PAN-GENs: (b) PVA-DSs/PAN-GENs 2% w/w PAN-GENs: (c) PVA-DSs/PAN-GENs 3% w/w PAN-GENs: (d) PVA-DSs/PAN-GENs 4% w/w PAN-GENs: (e) PVA-DSs/PAN-GENs 5% w/w PAN-GENs: (f). The high magnification of cell adhesion and cell migration for each sample sighed by the same character plus (^).

PVA and PAN polymers are well known biocompatible materials for fabricating nanofibers, but there was a concern that drug interactions with polymers may lead to the toxicity in contact with the wound. To determine the toxicity of the final product, samples were examined based on ISO 10993-5 and evaluated from grade zero (nontoxic) to grade four (severe toxic) in comparison to the positive (latex glove) and negative (absence of any sample) control^31^. As results are shown in Fig. 8, all samples showed the nontoxic behavior against fibroblast cells. The results showed that the cells grew well in the presence of core-shell nanofibers without any contamination due to the ineffectiveness of sterilization.

The cell proliferation has been don for evaluation of each sample in terms of long-term contact with the wound. The results, as shown in Fig. 8, emphasized the biocompatibility of samples after one day, but the number of cells significantly decreased during three days. However, the cells number changed to a stable situation when the medium culture was exchanged with the fresh one for follow up one-week cell proliferation. It seems that the high concentration of drugs or remaining the organic materials such as GA and DMF caused to reducing the cell proliferation during initial three days, but the situation was changed when the medium culture has been changed after three days. Also, cell migration and adhesion results showed that the morphology of cells was altered when cells adhered on the surface of 3% to 5% w/w PAN/GENs samples.

The results showed that prepared core-shell nanofibers meet the requirements for the preparation of appropriate wound dressing. While the cell adhesion occurred on the surface of all samples, it seems that for the chronic wound such as diabetic wounds, loading the grows factors may help to improve the cell adhesion and cell migration for better regeneration of wound.

### *In-vitro* release behavior

**Figure 9:**
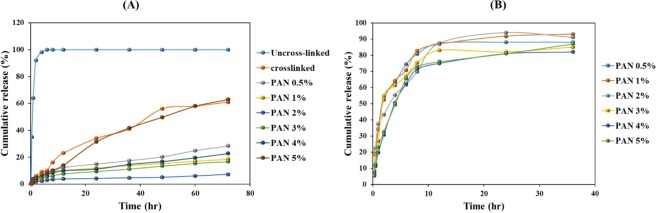
The drug release profile of DSs from nanofibers: (**A**) and GENs from nanofibers: (**B**). In all cases, n = 2 and SD <9%.

The nanofibers, when used for wound healing, need to evaluate drug release. Therefore, the produced core-shell nanofibers loaded by DSs/GENs with a ratio of 1:1 was studied for *in-vitro* drug release. As shown in Fig. 9(A), the results were indicated that the solubility of PVA nanofibers had a significant effect on the drug release. The results showed that the DSs release from PVA nanofibers was significantly decreased when PVA nanofibers were cross-linked via glutaraldehyde^32^. Also, the DSs release profile from PVA nanofibers after coating with PAN/GENs was significantly decreased due to two-stage release theory which introduced by S. Khansari *et al*.

Based on two-stage release theory, the PAN polymer containing the GENs is playing the substantial active role to control the DSs release from core nanofibers with covering the core part^33,34^. Therefore, the thickness of the shell had a significant effect on DSs release from the core and the nanofibers coated with PAN/GENs solution with higher concentrations in the core-shell structure caused to improve extended-release of DSs from PVA nanofibers.

On the other hand from the release profile related to GENs, as shown in Fig. 9(B), it was concluded that the PVA nanofibers coated by 0.5% to 2% w/w PAN/GENs polymer, had a faster release than nanofibers were coated with a high concentration of PAN/GENs solutions due to the high ratio of surface to area^35^.

The release of GENs has compared with previous studies. The results showed that the release of GENs had been completed after 12 hrs, that release duration was similar as reported for core-shell nanofibers in the current research^36^. On the other hand, the release from core nanofibers showed that DSs release was extended up to 60 hrs, while in the previous research the release time for DSs has been reported about 24 hrs when DSs had been loaded into the PVA nanofibers^37^.

The results showed that part of the drug was released during the washing process, but the core-shell nanofibers with concentrations of 1% to 2% PAN showed relevant results use as a carrier for drugs and current structure meet the demands to be used as local drug delivery systems for wound dressing.

## Conclusion

We are reported a facile method to obtain the advanced core-shell architecture for dual drug delivery system. The core-shell nanofibers were successfully designed and prepared for carrying both water-soluble and organic solvent soluble drugs at the same time. In comparison to traditional methods as well as co-axial electrospinning to obtain the core-shell nanofibers, this method was exhibited a high performance for a dual drug delivery system and wound dressing. Attributed to results the PAN concentration from 1% w/w up to 2% w/w were appropriate concentrations to achieve the unformed core-shell nanofibers. Prepared core-shell nanofibers showed the excellent biocompatibility in addition to optimized dual drug delivery, simultaneously. Also, it is believed that the method used in this study could extend to various drugs. Furthermore, this method could be the beginning of initial design for preparation of multi-layer nanofibers architecture for multiple drug delivery. Also, the prepared core-shell nanofibers showed the enormous potential of various fields such as protein delivery, sensors, photocatalysis, and tissue engineering in addition to drug delivery with changing the functionality instead of drugs.

## Experimental Section

### Materials

Sodium hydroxide with a purity of 97% (NaOH), acetone, ethanol (99.5), hydrochloric acid, purity of 35–37% and glutaraldehyde (GA) (50%) was purchased from Wako Chemical Industries, Ltd Japan. Gentamicin sulfate salt (GENs), diclofenac sodium salt, polyacrylonitrile (PAN) with (average Mw 150,000), polyvinyl alcohol (PVA), (Mw: 85,000–124,000, 87–89% hydrolyzed), Dimethylformamide (DMF) were purchased from Aldrich Chemical.

### Preparation of cross-linked PVA/DSs nanofibers

The PVA solution with a ratio of 11% w/w was prepared under stirring at 60 °C. The 5 mg/mL DSs was added to the obtained solution and mixed until a white suspension of PVA/DSs formed. The pH of viscose suspension was increased up to 9 by NaOH 0.5 M to obtain a transparent PVA/DSs solution. The clear PVA/DSs solution was subjected to a 20 mL syringe with a capillary tip gage of 20 with a 12 cm distance from the collector and supplying 17 kV, and continuously electrospinning continued for 24 hrs to obtain the PVA sheet nanofibers with the thickness of 0.16 mm. The viscosity of PVA polymer has been measured as 7.51 gr/cm/s with the surface tension of 39.85 mN/m and conductivity of 1.12 ms/cm.

The obtained PVA/DSs nanofibers mat was immersed for 10 minutes into the cross-linking solution prepared by a mixture of (GA): ethanol: hydrochloric acid (2:1:0.2 gm) in a 50 mL glass round bottom flask and the volume was adjusted to 50 mL by adding acetone. The unreacted HCl and GA washed out from cross-linked PVA/DSs nanofibers mat with deionized water for 5 minutes and dried at 60 °C for 30 minutes.

### Preparation of core-shell PVA/DSs-PAN/GENs nanofibers

The different concentrations of PAN solution (0.5%, 1%, 2%, 3%, 4%, 5%) were prepared in the DMF solution under stirring for 12 hrs, and 5 mg/mL GENs was added to the obtained solutions. The PVA/DSs nanofibers from the previous step were immersed in each PAN/GENs solution for 1 minute separately. The PVA/DSs nanofibers containing the PAN/GENs were immersed into the ethanol immediately for 30 seconds for crosslinking the PAN solution.

The final product was immersed in deionized water to wash out the ethanol and DMF for 10 minutes, and the obtained mat was dried at 37 °C for one hr. Figure 1 is showing the method in brief.

### Characterization

The Scanning Electron Microscope (SEM) (S-3000N, Hitachi Co., Japan) with an accelerating voltage of 15 kV has been employed for morphology observation of nanofibers after coating with platinum for 90 seconds. The Transmission Electron Microscope (TEM, 2010 Fas TEM, JEOL Japan) was conducted to observe the interface between core and shell nanofibers. Attenuated Total Reflectance (ATR) spectroscopy (DuraSamplIR II, Smiths Detection Company, United Kingdom) was used from 400 to 4000 cm^−1^. The thermal stability of each sample studied by the thermal- plus TG-8120 (Rigaku Corporation, Osaka, Japan) from 20 to 350 °C under air temperature at the heating rate of 10 °C. The toxicity of core-shell nanofibers was evaluated by using (ISO 10993-5). Briefly, the samples were sterilized by ethanol 70% and washed with PBS 1X for 5 minutes. The fibroblast cell from rat was obtained from Riken Bioresource Center (ID: RCB1862), Tsukuba, Japan and cultured in accordance with the guidelines set forth by the Riken Bioresource Center for the use for a cell culture test. The cells were cultured in 24 multi-well containing DMEM: FBS (85:15) until cells covered the 80% of the bottom at 37 °C with 5% CO_2_. The sterilized samples were placed on the cells layer and incubated for 24 hrs at the same conditions. In addition to toxicity evaluation, cell proliferation and cell adhesion assay were studied. The cell proliferation was evaluated via the WST-1 assay for viable cell percentage. Briefly, 1000 cell per well have been cultured in the presence of samples in the 96 multiwall plates and placed in an incubator at 37 °C for 1, 3, and 7 days respectively. After incubation, the WST-1 solution was added into each well with a ratio of 20% and incubate for 2 hrs. The plates were read at 560 nm with a microplate reader. For cell adhesion study, the cells were cultured for seven days in the presence of each sample, respectively. The cells fixed on the surface of samples, after washing with PBS and immersion into the glutaraldehyde 25% for 30 minutes. Dehydrate with ethanol series from 50% to ethanol absolute has been done for 2 hours and fixed cells were rated from nontoxic (grade 0) to severe toxically (grade 4), and samples have been used for morphology study via SEM. The Bunner-Emmett-Teller (BET) surface area of prepared samples was measured by using Micromeritics TriStar II 3020 version 3.02 with a degasser accessory Teledyne Hastings Instruments. The internal diameter of the sample tube was 3/8 inch, and 0.1 g of each sample was transferred in sample tubes, respectively. BET surface area was calculated by the amount of nitrogen adsorbed by the samples. The UV-visible Spectrophotometer (Lambda 900, Perkin-Elmer, USA) was employed to study the release behavior for 72 hrs. For this propose, the final core-shell nanofibers containing the models of drugs were immersed into the 20 mL deionized water under the shaking with a speed of 60 rpm. During the specified times (15 minutes, 30 minutes, 1 hr, 2hrs, 4hrs, 8 hrs, 12 hrs and then every 12 hrs) the 3 mL of solution from each sample was removed and exchanged with 3 mL fresh deionized water and kept in the refrigerator. The absorbance of removed solutions from each sample has been measured by UV-visible Spectrophotometer based on λ (max) for each drug. The λ (max) was measured 203 and 277 nm for gentamicin sulfate and diclofenac sodium salt, respectively. The amount of drug release compared with the standard curve and the cumulative release has been measured by the following formula:

$${\rm{Release}}( \% )=\frac{Mt}{Mi}\times 100$$

where Mt is the amount of drug released, and Mi is the total amount of drug loaded in each nanofiber
